# Towards sustainable human space exploration—priorities for radiation research to quantify and mitigate radiation risks

**DOI:** 10.1038/s41526-023-00262-7

**Published:** 2023-01-27

**Authors:** Anna Fogtman, Sarah Baatout, Bjorn Baselet, Thomas Berger, Christine E. Hellweg, Piers Jiggens, Chiara La Tessa, Livio Narici, Petteri Nieminen, Laure Sabatier, Giovanni Santin, Uwe Schneider, Ulrich Straube, Kevin Tabury, Walter Tinganelli, Linda Walsh, Marco Durante

**Affiliations:** 1 Space Applications Services for ESA - European Space Agency, Space Medicine Team and SciSpacE, HRE-RS, European Astronaut Centre (EAC), Linder Höhe, D-51147 Cologne, Germany; 2 Radiobiology Unit, Belgian Nuclear Research Centre, SCK CEN, Mol, Belgium; 3 DLR, German Aerospace Center, Institute of Aerospace Medicine, Linder Höhe, 51147 Cologne, Germany; 4 European Space Research and Technology Centre (ESTEC), Space Environment and Effects Section (TEC-EPS), Keplerlaan 1, 2201 Noordwijk, The Netherlands; 5 Department of Physics, University of Trento, Trento, Italy; 6 TIFPA, INFN, Trento, Italy; 7 Department of Physics, University of Rome Tor Vergata, 00133 Rome, Italy; 8 INFN – Section Roma2, Rome, Italy; 9 CEA/DRF/DIREI French Alternative Energies and Atomic Energy Commission (CEA), Paris-Saclay University, Gif sur Yvette Cedex, France; 10 Radiotherapy Hirslanden, Witellikerstrasse 40, 8032 Zurich, Switzerland; 11 Medical Operations and Space Medicine, HRE-OM, European Space Agency, ESA, European Astronaut Centre, EAC, Cologne, Germany; 12 Biophysics Department, GSI Helmholtzzentrum für Schwerionenforschung GmbH, Darmstadt, Germany; 13 Department of Physics, Science Faculty, University of Zürich, Winterthurerstrasse 190, 8057 Zurich, Switzerland; 14 Technische Universität Darmstadt, Institute for Condensed Matter Physics, Darmstadt, Germany; 15 Universita‘ Federico II, Dipartimento di Fisica “Ettore Pancini”, Naples, Italy

**Keywords:** Risk factors, Biophysics

## Abstract

Human spaceflight is entering a new era of sustainable human space exploration. By 2030 humans will regularly fly to the Moon’s orbit, return to the Moon’s surface and preparations for crewed Mars missions will intensify. In planning these undertakings, several challenges will need to be addressed in order to ensure the safety of astronauts during their space travels. One of the important challenges to overcome, that could be a major showstopper of the space endeavor, is the exposure to the space radiation environment. There is an urgent need for quantifying, managing and limiting the detrimental health risks and electronics damage induced by space radiation exposure. Such risks raise key priority topics for space research programs. Risk limitation involves obtaining a better understanding of space weather phenomena and the complex radiation environment in spaceflight, as well as developing and applying accurate dosimetric instruments, understanding related short- and long-term health risks, and strategies for effective countermeasures to minimize both exposure to space radiation and the remaining effects post exposure. The ESA/SciSpacE Space Radiation White Paper identifies those topics and underlines priorities for future research and development, to enable safe human and robotic exploration of space beyond Low Earth Orbit.

## Introduction

International space agencies are entering a new phase of sustainable human and robotic exploration of space Beyond Low Earth Orbit (BLEO). Recent advances in construction of the Moon-orbiting Gateway station bring those plans closer to realization, with new scientific, engineering, and operational challenges ahead. In comparison with the current mission profiles to the International Space Station (ISS), the first set of missions to the Gateway and Moon surface will be shorter (30–90 days), there will be less habitable space (~10 fold) for astronauts, smaller payloads, a slight delay in communication and no possibility of a quick emergency return to Earth. Such conditions will form the basis of sustainable human presence in deep space, involving months to years of exposure to space hazards.

The complexity of the radiation environment in deep space adds complexity to the overall risk assessment for human BLEO spaceflight, and the recently published Radiation White Paper^1^ by the European Space Agency (ESA) assesses such challenges. The radiation environment in deep space differs substantially from the conditions in Low Earth Orbit (LEO), where astronauts are—at least partly—shielded from the complex spectrum of particles and energies by Earth’s magnetosphere. In deep space, astronauts will be exposed to Galactic Cosmic Rays (GCR) composed of protons, helium ions and rarer but highly energetic (up to and exceeding 100 GeV per nucleon) heavier nuclei^2^. Astronauts may also be exposed to sporadic radiation storms originating from solar eruptions with particle energies at energies <1 GeV/n, known as Solar Particle Events (SPEs), which can result in considerable dose accumulation in case of insufficient shielding. These particles will interact with spacecraft shielding, payload, space suits, and planetary or Moon regolith, to create a cascade of secondary particles, where neutrons may also play a significant role, especially for thick shielding and surface habitat scenarios. Ionizing radiation (IR) in deep space can be a single limiting factor to human space exploration. To fly safely, space agencies need to be able to assess the radiation space environment, reduce the exposure, predict the health risks and mitigate the negative effects of exposure to IR. Gaining those capabilities is an interdisciplinary endeavor, involving space weather assessment and predictions, shielding, dosimetry, radiobiology, radiation epidemiology, risk assessments, and space medicine.

## Radiation measurements and simulations

**Fig. 1:**
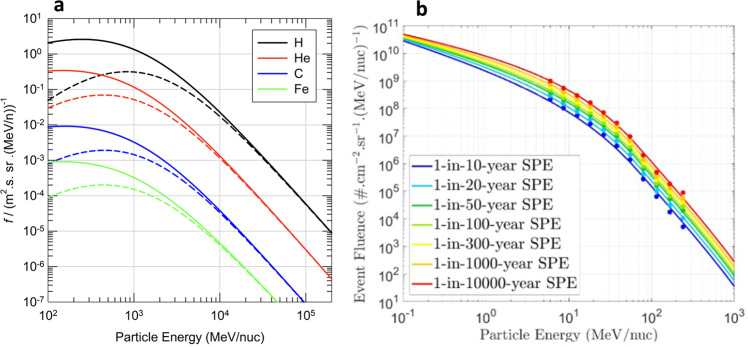
Energy spectrum of space radiation. **a** The galactic cosmic radiation spectrum in free space for solar maximum (dashed line) and solar minimum (solid line) conditions as calculated with the DLR GCR Model^47^ for four different ions. Figure from the DLR image database **b** The solar energetic particle radiation fluence environment in free space for rare “extreme” events as calculated with the SAPPHIRE Model^48^. The model shows that more intense and energetic events are more seldom (the lines correspond to events occurring with a mean frequency of 1-in-N years). Figure produced by ESA-ESTEC using the SAPPHIRE-network of models server.

Understanding space radiation risk for humans requires a precise knowledge of the radiation field in space, the possibility to calculate the radiation exposure in different scenarios, and appropriate models to assess the relevant risks. For BLEO missions, GCR and SPEs (Fig. 1) will provide a more severe radiation environment compared to the more protected missions onboard the ISS.

The relevant questions to be answered would therefore fall into the following categories.

1. Radiation environment measurement
  For mission planning and design, the radiation environment must be specified to ensure that humans and electronics will be able to withstand the environment being protected by the shielding provided by the spacecraft/habitat. This requires the use of newly developed instrumentation, capable of providing the relevant information and also the analysis of data from existing detector systems, as on the surface of the Moon^3^ or on other locations^4,5^, to intercompare and intercalibrate the new devices data.
2. Dosimetry and radiation risk estimation
  Personal dosimetry^6^ should be regarded as separate from the radiation environment measurements being directly related to the operational radiation protection of the astronauts fulfilling the requirements given by the space agencies. For exploration missions a paradigm change has to be implemented in providing actively powered radiation detectors for the crew, which not only enable “real time dose readings” but could also provide required physical parameters for risk estimation. Individual biodosimetry^7^ needs to be implemented and compared with the results of radiation detectors in order to contribute to risk estimate. Indeed, the biological dosimetry of astronauts from the same space mission can differ according to their own individual radiosensitivity and their own activities during the mission (EVAs, location in the station during SPEs,…).
3. Radiation propagation tools and models
  Radiation propagation tools provide significant calculated radiation data for a specific planned mission scenario and can be benchmarked with data from sensors measuring relevant parameters in new environments^8^. GCR and SPE models also demand a detailed benchmarking against each other and against measurements. In addition, transport codes, based on Monte Carlo (GEANT4, FLUKA, PHITS) or on deterministic (HZETRN) codes need further developments including updates for missing data in nuclear cross section measurements^9^.
4. Radiation storm forecasting.

SPEs are a manifestation of space weather^10^ giving rise to drastically enhanced radiation levels in a short time. Forecasting SPEs is very important for BLEO operations for efficient planning and use of countermeasures complying with the ALARA principle for astronaut protection. SPE forecasting utilizes knowledge of solar physics and particle radiation dynamics either explicitly by radiation transport in the case of physics-based models or implicitly in the case of analytical models^11^. Forecasting can be triggered by solar observations or by in-situ measurements outside the human habitat (vessel, base) of SPE precursor radiation (now-casting). Presently, forecasting from physics-based models lacks the accuracy needed for human protection. Now-casting, based on precursor measurements combined with studies of previous SPEs, are essential for effective warnings^12^.

Development priorities include a system to exploit forecast methods and accurate measurements of the external field for now-casting and data assimilative forecasts. Eventually, forecasts based on solar physics and particle transport models will provide improved performance.

## Radiation risks

**Fig. 2:**
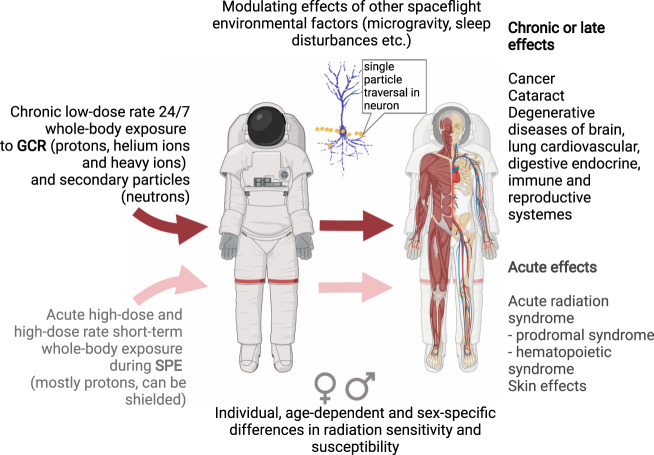
Radiation exposure during space missions beyond low Earth orbit and health effects of space radiation. Illustration created with BioRender.com for this paper.

During deep space exploration, astronauts experience a chronic, low-dose-rate whole-body exposure to GCR (Fig. 2), which can accrue to ~1 Sv during a 1000-day Mars mission^13–15^. Due to the physical properties of the particle radiation and the heavy ion component of the exposure, high doses can be reached at a microscopic level, resulting in complex, difficult to repair DNA damages. Unrepaired or misrepaired DNA lesions are responsible for cell death and mutations and eventually late effects such as cancer^16^ or normal tissue degenerative processes including cardiovascular disease^17^ or central nervous system (CNS) damage^18^. Increased cancer risk is the endpoint generally considered in assessing the lifetime exposure limits in LEO^19,20^. The attempts to understand the space radiation-induced cancer risk encompass multiple levels:

1. Cancer mortality studies among astronauts: The cohort is small, and the current studies cover mostly short missions in LEO and the Apollo missions. The long latency periods, low statistics, and low doses preclude currently the assessment of the effect of the ISS missions of ~ half a year duration. Furthermore, a strong healthy worker effect^21^ was observed in the NASA Longitudinal Study of Astronaut Health, masking possible radiation-induced cancer risks^22^. In fact, even if there is an apparent increase in melanoma and prostate cancer, this is likely to result from increased cancer screening.
2. Exposure of animals (wildtype and genetically altered rodent models, mostly mice) to space-relevant radiation qualities experiments and follow-up of cancer induction, and mechanistic studies at molecular, cellular, tissue, organ, or organismal level. The relative biological effectiveness (RBE) for cancer induction by a specific GCR component, e.g., iron or silicon ions, can be the result of such studies. Such RBE values are helpful to extrapolate cancer risk from larger radiation-exposed populations (the atomic bomb survivors are the most prominent example) which experienced acute radiation exposure (mostly γ-rays, also neutrons) to GCR-exposed astronauts. Rather than studying single ions it is now possible to simulate the full GCR spectrum on Earth both in USA^23^ and European^24^ accelerators and this will pave the way to realistic RBE estimation of the full GCR. In addition, experiments to quantify the effect of low dose rates as expected for GCR-exposure compared to the high-dose rate exposure by the atomic bombs are performed to determine the dose-rate reduction factor, but these experiments are almost impossible at particle accelerators as they would require long exposure times. The uncertainties of cancer risk assessment are still unacceptably high^25^.
3. Spaceflight experiments using different biological models help to clarify the role of other space environmental factors such as microgravity in the modulation of GCR-induced cancer risk.

A cell hit by an energetic particle experiences DNA damage that might be mis- or unrepaired as well as induce changes in gene expression depending on the dose and the linear energy transfer of the heavy ions^26^. These changes can be perpetuated by epigenetic alterations, high oxidative stress levels^27^ and senescence. Damage can be transmitted in the progeny of irradiated cells and chromosomal instability can occur^28^. These might contribute to carcinogenesis and could be the basis for late degenerative processes in several organs. Currently, CNS, eye lens, lung, cardiovascular, digestive, endocrine and immune systems, and the reproductive organs are considered to be at risk for space radiation-induced degenerative processes. Calculations suggest that for a three-year mission to Mars at a solar minimum, 2–13% of the “critical sites” of cells in the CNS would be directly hit at least once by iron ions, and roughly 20 million out of 43 million hippocampal cells and 230,000 out of 1.8 million thalamus cell nuclei would be directly hit by one or more particles with Z > 15 on such a mission^29^—in combination with the extremely low regenerative potential of the brain, this is a reason for concern. Also, earlier or more frequent cataract formation was observed in astronauts on higher inclination LEO missions^30^. The RBEs of heavy ions for these endpoints are scarcely known. Further accelerator-based studies are required to include the risk of degenerative diseases in the space radiation risk assessment.

In case of a large, unpredicted SPEs and a low shielding situation (e.g., during an EVA), astronauts can experience an acute whole-body exposure to energetic protons and accumulate high skin doses and effective doses of ~2 Gy within several hours or days. In this dose range, acute radiation syndrome with the hematopoietic system as main target can be expected^31^. Such exposures have to be prevented by the space weather forecast, nowcast, active dosimetry, and appropriate shielding. SPEs, therefore, mainly represent an operational medical problem, but can contribute to the late health risk if shielding does not reduce the accumulated dose to a negligible level.

## Risk estimations

The above-mentioned health risks need to be understood and assessed, in order to predict the frequency and latency of the late effects. The development of ESA radiation risk models, to better characterize the mission radiation risks to astronauts, was recently recommended in a paper on research plans in Europe for radiation hazard assessments in space^19^. In line with this recommendation and the radiation protection initiative for astronauts at the ESA-Astronaut Centre, the first stage of a space radiation risk module for Astronaut’s health risk assessment was developed and verified^32^. This risk module built on previous work^33,34^ was based on radiation-related health risk assessment for the detrimental health effect outcomes of incidence of all solid cancer, leukemia, lung and female breast cancer from estimated radiation exposures accumulated during long term missions to the Moon or Mars. An alternative approach based on the quantity called Radiation Attributed Decrease of Survival (RADS)^35^ was proposed. RADS represents the cumulative decrease in the unknown survival curve at a certain attained age, due to the radiation exposure at an earlier age.

Applying this approach, a 1000-day Mars exploration mission with a hypothetical effective dose of ~1 Sv received at a typical astronaut age of 40 years old, was found to result in the probability of surviving free of all types of solid cancer and leukemia until retirement age (65 years) being reduced by 4.2% (95%CI: 3.2; 5.3) for males and 5.8% (95%CI: 4.8; 7.0) for females^32^.

Recommendations in a recent National Academies of Sciences, Engineering, and Medicine report^36^ connected with communicating a comprehensive picture of an individual astronaut’s cancer risks due to radiation exposure and examining the application of risk metrics other than risk of exposure-induced death are fully in line with the new ESA approach. Furthermore, the ESA module has a more comprehensive uncertainty assessment than the current NASA Space Cancer Risk Model (NSCR). In the NSCR, the “tissue specific statistical error” represents subjectively chosen uncertainties only in the central estimate of the sex-specific main radiation risk to dose response, i.e., excess risk per Sv, β. Uncertainties associated with attained age and age at exposure risk effect modifiers of β are not explicitly accounted for^20^. In contrast, the ESA module fully accounts for the published uncertainties in β and the uncertainties on the attained age and age at exposure risk effect modifiers via Monte-Carlo simulation, considering all the correlations between these quantities (see e.g.,^34,36^).

Further work which builds on and extends this form of risk assessment approach is currently underway at ESA to examine the feasibility of eventually including non-cancer effects and to include organ doses from detailed astronaut space dosimetry. Those risk estimates are modified by mitigation strategies including both, physical and biological countermeasures.

## Risk mitigation: physics

As the risk is directly related to the dose, a mitigation strategy has to be developed to decrease it to an acceptable level. The dose depends on the mission scenario, namely the type of radiation field to which the astronauts are exposed to, as well as on the duration, and therefore it can span over a wide range of values. For example, the average dose received by astronauts inside the ISS is around 0.5–1 mSv/day^37^, while for the Mars mission the RAD instrument (onboard the unmanned MSL “Curiosity”) measured in free space an averaged GCR dose equivalent rate of 1.84 mSv/day^14^, and the equivalent estimate for the Martian surface is 0.64 mSv/day. Based on these dose rates, which are mainly delivered by GCR and can be further increased by potential SPEs, the current estimates for a full Mars mission are critically high, exceeding most space agency limits that are set at 0.6–1 Sv^38^.

Physics-based mitigation methods aim at decreasing the dose by acting on the incoming radiation field in two different ways: i) active shielding deflecting particles with magnetic or electrostatic fields, and ii) passive shielding, exploiting nuclear and electromagnetic interactions between the incoming radiation and materials^39^. While active shielding is promising and still an active field of research, it is in a very preliminary phase^40^, and it is unclear whether a realistic active solution could provide adequate protection against the high-energy GCR component of the space radiation field. As a result, today the only countermeasure applied in space radioprotection is passive shielding and limiting permissible mission duration. Although it would be ideal to employ a shield that completely stops all external radiation, this cannot be achieved because of the mass load constraints of spacecraft designs. For this reason, the approach of space shielding is based on decreasing the dose by modifying the radiation field composition via nuclear fragmentation, namely by breaking ions into particles of lower charge and similar velocity, while still avoiding dose enhancements from the resulting mixed field of secondary particles.

Over the years, this method has experienced a paradigm-shift, evolving from dedicated shields added to the spacecraft, to the concept of designing the actual spacecraft with multifunctional elements, optimized both for their primary use and for their shielding effectiveness. On Moon and planetary surface habitats, this method can also be complemented by use of local in-situ resource utilization, by placing the structures underneath thick layers of regolith.

Dedicated shielding materials can also be added to the structure to further decrease the environment dose, and their design is optimized depending on the mission scenario. In this framework, ESA has been supporting theoretical and experimental studies on space radiation shielding (ROSSINI)^41^, aiming at dose reduction through optimization of structure configuration and research into innovative materials. These studies identified lithium hydride (LiH) compounds as a promising alternative to polyethylene, which is currently used on the ISS as radiation shielding^42^.

## Risk mitigation: biology

From an evolutionary perspective there seems to be no trait that enabled eukaryotic organisms to survive IR doses in the range to which several extremophiles are capable of surviving. However, ancestors of the modern human all evolved in an environment consisting of a persistent low level of different mutagenic agents. As a consequence, we have many inherent cellular mechanisms to counteract DNA damage and oxidative stress. Yet, when humans travel into space, these naturally evolved cellular mechanisms are not enough as morbidities resulting from space radiation exposure have been identified (e.g., cataract and immune dysfunction). In order to support future deep space exploration missions, possible interventions can be conceived that can limit the effects of space radiation on the human body and as a result can reduce the health risk in humans when exposed to space.

**Fig. 3:**
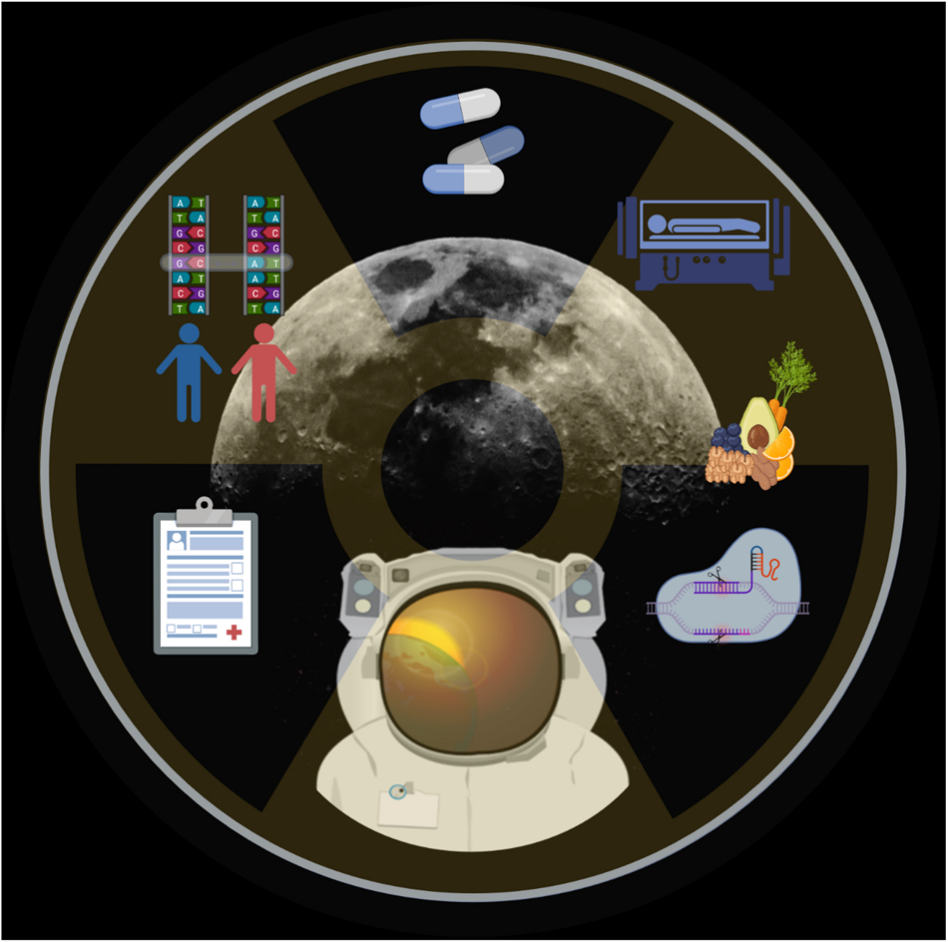
Principles of interventions to reduce health risk from space radiation exposure (clockwise from lower left). Selection campaigns—genome-wide association studies—radioprotective pharmaceuticals—hibernation (synthetic torpor)^49^—food supplements—genome editing. Illustration created by the authors for this manuscript.

So far, six principal interventions have been proposed to reduce the health risk from space radiation exposure (Fig. 3).

One way of reducing the health risk from space radiation exposure in humans is selecting for more radioresistant humans during the selection campaigns of space agencies. It is in fact known that susceptibility to radiogenic late effects presents a wide inter-individual variability and this also applies to space radiation exposure^43^. The simplest approach is to perform ex vivo assays, in which cells collected from the candidates are exposed to a fixed IR dose^44^. In addition, genome-wide association studies to determine the single nucleotide polymorphism (SNP) and epigenetic profiles of radioresistant individuals could also be used. Before practical applications, however, it will be necessary to establish a link between genetic profiles and cancer (or other late effects) susceptibility. Whilst capabilities to identify SNPs correlated to specific normal tissue toxicities after radiotherapy have been significantly advanced^45^, a lot of progress has to be done concerning radiogenic cancers and very late effects. Another strategy is to pharmacologically hamper with the processes underlying the molecular (side) effects of space radiation exposure. Examples are the application of radioprotectors and geroprotectors, as well as supplementation with antioxidants or anti-oxidative capacity increasing compounds. While these pharmaceuticals hold great promise, many of them are still under investigation and not allowed to be used on humans.

Food supplements (such as vitamin A, C, D, omega, selenium, antioxidants (polyphenols)) to boost the immune system, have an anti-ageing effect, and reduce oxidative stress is another strategy. Finally, through the avenues of gene editing, modification of the human genome becomes a possibility, especially CRISPR-based tools to modify gene expression without modifying the DNA sequence^46^. Promising strategies are the inducible expression of endogenous antioxidants, DNA repair genes or radioprotective transgenes resulting in controlled reduction in early and late-stage irradiation damage^44^. Ethically, these genetic modifications remain under debate. Altogether, mitigation risks for future deep space exploration missions are currently under investigation as they appear to bring promising solutions.

## Outlook and summary

Future deep space exploration involving long and sustainable human presence in space requires a state-of-the-art approach to protect astronauts from the detrimental effects of the space radiation environment. It is a priority to build a robust, reliable, and comprehensive system for astronaut radiation protection. The Space Radiation white paper identifies key topics to guide ESA/SciSpacE research programmes and in consequence—build European expertise to help bring ESA and international astronauts safely to the Moon and Mars. These space challenges are also of paramount concern for humans on Earth, as IR is a risk factor in many sectors, including public health and energy. Therefore, a multidisciplinary approach needs to be taken to address those challenges to further advance capabilities:

1. Understanding the space radiation environment outside and inside spacecraft, landers and habitats with the use of appropriate instruments capturing the IR spectrum in space most important for protection of humans and electronics, and models of interaction of IR with physical matter.
2. Predicting the space weather with the use of forecasting models and accurate measurements of the space radiation environment and observations of the Sun.
3. Understanding the health effects of long-term exposure to low dose rates of complex-spectrum of IR, by studying scarce astronaut cohorts, as well as spaceflight and particle accelerator-based studies on animals and cell cultures with space-relevant radiation qualities of IR.
4. Accurately predicting health risks from exposure, with the use of mathematical models based on epidemiological and experimental data at accelerators.
5. Mitigating the health risks with utilization of shielding approaches effective for GCR and SPEs, as well as assessment of individual susceptibility to IR and use of biological and pharmacological countermeasures.

As often noted previously, insufficient knowledge on biological mechanisms and effects, especially those related to how and to what extent very heavy ions interact with and damage human tissue, account for the largest fraction of uncertainty in IR related health risk assessment. These challenges must be addressed by European and international space programmes, with close collaboration between applied sciences and medical operations, to enable faster and affordable certification processes of developed hardware and medications. Significant budgets will have to be dedicated to space radiation research enabling exploration, and agencies will need to increase the visibility of their space radiation programmes to draw new talents and innovative ideas to this critical problem for space exploration. Tackling the above points is crucial to enable a safe and sustainable human presence in deep space.
